# A Combined Dietary and Cognitive Intervention in 3–5-Year-Old Children in Indonesia: A Randomized Controlled Trial

**DOI:** 10.3390/nu10101394

**Published:** 2018-10-01

**Authors:** Nora Schneider, Eveline Geiser, Laura M. Gosoniu, Yulianti Wibowo, Gertrude Gentile-Rapinett, Mayke S. Tedjasaputra, Sudigdo Sastroasmoro

**Affiliations:** 1 Nestec Ltd., Nestlé Research Center, 1000 Lausanne 26, Switzerland; eveline.geiser@rd.nestle.com (E.G.); marialaura.gosoniu@rdls.nestle.com (L.M.G.); Gertrude.Gentile-Rapinett@rdls.nestle.com (G.G.-R.); 2 Nestlé Indonesia, Nestlé Nutrition Institute, TB Simatupang kav 88 Jakarta Selatan, Jakarta 12520, Indonesia; Yulianti.Wibowo@id.nestle.com; 3 Faculty of Psychology, University of Indonesia, Kampus UI, Depok 16424, Indonesia; maykeui@gmail.com; 4 Faculty of Medicine, University of Indonesia-Department of Child Health Cipto Mangunkusumo Hospital, Jakarta Pusat 10430, Indonesia; s_sudigdo@yahoo.com

**Keywords:** growing up milk, fortification, cognitive stimulation, cognitive development

## Abstract

Early childhood nutritional interventions typically combine nutritional and psychosocial stimulation. Such combined interventions result in long-lasting improvements of cognitive abilities in children who are malnourished. Here, we investigated potential cognitive abilities in normally developing children in Indonesia who were, however, at risk for suboptimal cognitive development due to little psychosocial stimulation in their home environment. In a randomized controlled intervention, children of the experimental group received nutritional supplementation combined with cognitive stimulation. Pre- and post-intervention measurements included cognitive development and functioning, behavior, and mother–child interaction. The experimental and control group received nutritional supplementation in the form of a fortified or unfortified milk powder, respectively. Additionally, the children and parents of the experimental group jointly engaged in daily learning activities at home and performed iPad-based tasks designed to foster cognitive abilities. The experimental group compared to the control group displayed a significantly higher increase in intelligence quotient as well as a significantly larger reduction in attentional problems after the intervention. These results indicate that low-level cognitive stimulation in combination with nutritional supplementation during early childhood can be an effective intervention that improves global cognitive functioning in healthy developing children. ClinicalTrials.gov Identifier: NCT02359669.

## 1. Introduction

The development of a child depends on a range of mutually interacting factors including genetic, individual (e.g., temperament and personality), biological (e.g., health and nutritional status), environmental (e.g., stimulation, quality of mother–child interaction), and cultural (access to education) influences. Specifically, healthy cognitive development draws, among other factors, on psychosocial stimulation and sufficient nutrition (for a review, see [1]). For example, almost half of the variance (43%) in cognitive development in 3–5-year-old children (*n* = 58) coming from a heterogeneous socioeconomic background in Indonesia can be explained by psychosocial stimulation, education, and nutritional status [2]. In developing countries, often both cognitive stimulation and nutrition are lacking, resulting in an estimated 200 million children under the age of five worldwide not reaching their potential for cognitive developmental [3,4]. Early childhood intervention programs comprising an educational or stimulation component typically show larger effects on cognition than nutritional interventions alone [5,6]. Furthermore, a series of intervention studies in developing countries that report benefits on cognition combined a nutritional intervention with psychosocial stimulation (for reviews and meta-analyses, see [7,8,9]). In a study in Bangladesh, a nutritional supplementation by means of a balanced meal combined with advice on child development and mother–child interaction in 6–24-month-old children (*n* = 193) resulted in improved mental development [10]. Similarly, a study in Mozambique paired psychosocial stimulation with nutritional advice given to parents and demonstrated positive effects in cognitive, motor, and emotional domains in the intervention group (*n* = 107) compared to the control group (*n* = 107) [11]. Comparable effects were observed in studies in Bolivia [12] and Columbia [13,14,15]. Thus, successful intervention programs focusing on cognitive development often combine psychosocial with nutritional stimulation.

Most early childhood intervention studies that combine nutritional and psychosocial stimulation report long-lasting effects on cognition that sometimes last into adolescence. A study that provided nutritional supplementation and/or psychosocial stimulation to growth-restricted Jamaican children (*n* = 129) at preschool age yielded advantages in mental development after the intervention [16] and also showed that benefits to cognitive functioning persisted in these children (*n* = 127) into school age [17]. When children (*n* = 116) reached 11 and 12 years, the effect of nutritional supplementation in the form of milk-based formula alone had ceased while children who had received stimulation at preschool age had higher scores on the Wechsler Intelligence Scales for full-scale IQ, verbal scale, and vocabulary [4]. Similarly, two studies conducted in Vietnam and Pakistan reported long-lasting positive effects of the psychosocial intervention aspect on cognition 2–4 years after an early childhood intervention [18,19]. Only one of the previously mentioned studies, performed in Columbia including 399 children, did not find a positive long-term effect 3 years after the intervention [14]. Overall cognitive benefits of early childhood interventions involving nutritional and psychosocial stimulation often persist over time and, namely, the psychosocial components of interventions can last at least into adolescence.

Psychosocial intervention programs have adopted a variety of intervention methods. These include the education of caregivers [10,18,19,20] but also child-focused interventions such providing professional care at childcare centers [15,21]. A study in Turkey compared different modes of interventions in 127 children and found that both child-focused and parent-focused interventions resulted in positive cognitive effects, with parent-based interventions yielding longer lasting benefits, namely, on school grades and verbal scores [21]. The best effects, however, seem to come from programs integrating both a parent and a child-focused intervention component [20]. Thus, current evidence indicates that an optimal psychosocial stimulation should integrate both enrichment programs for children as well as focus on the involvement of parents in the cognitive development of their children.

The above evidence indicates that childhood interventions at preschool age have a significant impact on the lives of children and yield positive effects, namely, on their cognitive abilities. However, most previous studies focused on children that were malnourished and/or stunted at baseline. Therefore, the impact of such intervention in normally developing children is unknown. Here, we assessed the effect of a nutritional intervention combined with a low-level psychosocial stimulation on cognitive abilities of healthy developing preschool-aged Indonesian children who were raised in homes with low psychosocial stimulation and low educational background as measured by the Home Observation for Measurement of the Environment (HOME) Inventory [22]. Children raised in homes with low measures in the HOME Inventory are known to display lower levels of inhibitory control and cognitive flexibility [23], decreased attentional control [24], and decreased working memory and planning capacities [25], all of which result in reduced overall cognitive abilities. Children of the experimental group received daily nutritional supplementation and performed iPad-based games designed to support cognitive and brain development. Games were played in interaction with their caregiver. Additional paper–pencil learning materials were used at home. In a randomized controlled study design, cognitive abilities in the experimental and control group were assessed at baseline and at the end of the 6-month intervention. To our knowledge, this child-focused intervention study is the first to provide digital tasks fostering cognition in cognitively normally developing preschool-aged children. We hypothesized that the cognitive abilities of the children in the experimental group compared to the control group would increase during the intervention.

## 2. Materials and Methods

### 2.1. Participants

Children were recruited in 2014 from the general population in Jakarta, Indonesia by means of advertisement through volunteers working in community health centers. To ensure unbiased screening, all 460 children who showed interested in the study were invited to an initial screening visit. Children were enrolled in the study if they fulfilled all inclusion criteria and presented none of the exclusion criteria. The following inclusion criteria were applied: (1) A below-average level of stimulation in the home environment indicated by a score below 26 in the HOME Inventory. This corresponds to a stimulation level 1.73 SD below the population mean [26]. This inventory was used in previous studies focusing on the same geographic region and age range [2,27]. We adjusted the cutoff to reflect high stimulation levels observed in the tested population; (2) An age between 3 and 5 years; (3) Normal or above normal cognitive development according to the DIKNAS test of the National Department of Education of Indonesia (http://www.kemdiknas.go.id/); and (4) A weight for height within 2 SD from the median z-score as implemented using WHO Anthro software (version 3.2.2, http://www.who.int/childgrowth/software/en/). We excluded children if they had a history of neurological disorders, food allergies, documented lactose intolerance, special nutritional needs, chronic medical conditions requiring medication for more than 7 consecutive days potentially impacting cognitive performance, lived in foster homes, were not expected to comply with the study procedures, have been participating in another clinical study within 4 weeks prior to the beginning of the study, or if their parents were not able to read sufficiently. Out of the selected children, 200 children were enrolled in the study and randomly assigned to either the experimental or the control group. According to the power analyses performed at the protocol stage, 128 subjects completing the 6-month intervention were needed in order to detect a statistically significant difference of 7 points in the Wechsler Preschool and Primary Scale of Intelligence (WPPSI) score between the two groups (standard deviation 14) with a 80% statistical power and 5% type I error. To allow for a 35% drop-out rate, 200 subjects were recruited. The full analysis set consisted of 192 children (mean age 3.92 ± 0.5 (SD) years, 102 boys) assigned to either the experimental group (*n* = 95) or the control group (*n* = 97). The study was performed at four research sites in Jakarta and in accordance with the ethical guidelines of the Declaration of Helsinki and approved by the Ethics Committee of the Faculty of Medicine, University of Indonesia (‘34/H2.F1/ETIK/2013’ and ‘492/H2.F1/Etik/VII/2014’). Written informed consent was obtained from the legal representative of each child.

### 2.2. Experimental Design and Procedure

**Figure 1:**
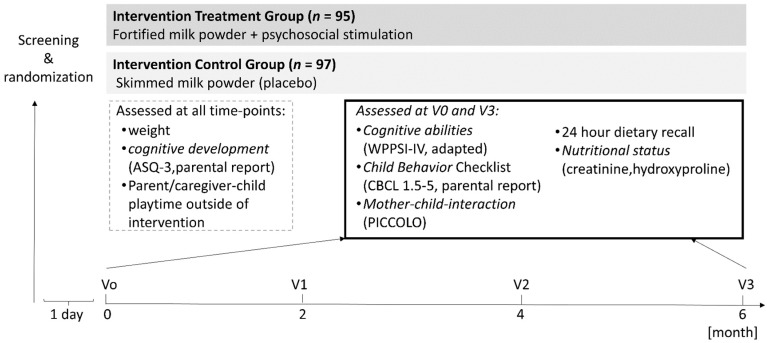
Design of the randomized intervention study and summary of assessments taken at all time-points and at baseline and V_3_, respectively.

The study adopted a parallel, randomized clinical trial with an intervention period of 6 months and included an experimental (*n* = 95) and a control (*n* = 97) group. The experimental group consumed at home two servings of a fortified milk powder and performed cognitive stimulation tasks at least 3 days per week (average duration 32 min per week). The control group consumed at home two servings of unfortified skimmed milk powder and received no additional cognitive stimulation tasks. One serving of the fortified or skimmed milk powder, respectively, consisted of 36 g of powder diluted in 180 mL of warm water. The two milk powders were isocaloric. Fortification consisted of polyunsaturated fatty acids, vitamins, and minerals that were previously associated with cognitive performance in children ([28,29,30,31], Table 1). Dosage of nutrients was in accordance with the regulations of the National Agency of Drug and Food Control of Indonesia. After screening for eligibility, children were randomly assigned to either the experimental or the control group. The assignment was nonobvious to the experimenter and controlled for gender and age using a minimization method [32] implemented in Medidata Balance (https://www.mdsol.com/en/products/rave/rtsm). Assessment of outcome measures for all children took place at baseline (V_0_) as well as after the 6-month intervention (V_3_). Additional testing was performed at 2 (V_1_) and 4 (V_2_) months (±1 week) after the start of the intervention (Figure 1). Psychologists assessing cognitive outcome variables were blind to the experimental groups, not involved in the random assignment of the children, and not involved in the psychosocial stimulation sessions. Children and caregivers were blind to the nature of the nutritional intervention.

### 2.3. Psychosocial Stimulation

Stimulation of the experimental group was twofold. Children performed five different tasks on an iPad application designed to train memory, language, psychomotor skills, problem solving, and attention (https://www.stimulearn.com/en). Specifically, each of the games involved either reading, counting, naming items, memory tasks, puzzles, and moving object detection. The children could choose between several levels of difficulty and decided themselves how long they spent performing each task. Moreover, the stimulation was designed to foster parent–child interaction in that the tasks were performed with the assistance of the parent/caregiver. The iPad application was provided to children in the community center and participants were instructed to come to the center for 10–15 min for 3–5 days per week. A trained psychologist assisted in the performance of the tasks. Additionally, standardized learning activities targeted at enhancing communication, gross motor, fine motor, problem solving, and personal–social development were offered to parents for use with their children at home (Ages and Stages Questionnaire (ASQ) Learning Activities; [33]). This material was distributed in the form of a book and included suggestions to the parents on how to play with the child such as playing with water, cooking, or drawing. The children of the control group did not come to the community center and did not receive any psychosocial stimulation.

### 2.4. Cognitive, Behavioral, and Nutritional Assessments

A list of tests used to assess cognitive, behavioral, and nutritional variables is provided in Figure 1 and described in this section. No primary and secondary outcomes were defined as this was an exploratory trial. We assessed *cognitive functioning* by the Wechsler Preschool and Primary Scale of Intelligence, 4th edition (WPPSI-IV), a standardized test of cognitive abilities in 2.5–7.5-year-old children including measures of verbal comprehension, visual spatial processing, working memory, fluid reasoning, and processing speed. For this purpose, a back and forth translation of the English WPPSI-IV to Bahasa Indonesia was completed before the study and feasibility as well as understanding of the test material were tested in a pilot study comprising 40 children [34,35,36]. After validation, a few test items were revised to accommodate cultural specificities. Composite scores were analyzed for group differences. *Cognitive development* was assessed by the Ages and Stages Questionnaire, 3rd edition (ASQ-3) including measures of communication, gross and fine motor skills, problem solving, and personal–social interaction (http://www.brookespublishing.com/resource-center/screening-and-assessment/asq/asq-3/). *Mother–child-interaction* was assessed by means of the Parenting Interactions with Children: Checklist of Observations Linked to Outcomes (PICCOLO) checklist covering measures of affection, responsiveness, encouragement, and teaching. The rating was based on 10-min videotaped sequences. *Child behavior* was assessed through parental rating by means of the Child Behavior Checklist for 1.5–5 years (CBCL 1.5–5) assessing emotionally reactive behavior, anxious/depressed behavior, somatic complaints, withdrawn behavior, attention problems, aggressive behavior, and sleep problems (http://www.aseba.org/preschool.html). Additionally, *stimulation/play time of the parent with the child* outside of the intervention, motivation, and other major life events were assessed by means of a custom-made parental diary.

We assessed dietary intake by a 1-day, 24-h dietary recall. The investigator interviewed the primary caregiver of the children and entered the information into the software NutriSurvey (http://www.nutrisurvey.de/). This software incorporates a dietary food composition table that translates the amount of different foods reported into an amount of macro- and micronutrients. Nutritional status and growth were assessed by means of anthropometric measures (weight, height) and the Hydroxyproline Index measured in urine samples [37].

### 2.5. Analysis

The statistical analysis plan was defined in a blinded manner before breaking the code. The full analysis set (experimental group: *n* = 95, control group: *n* = 97) was considered the primary analysis dataset and consisted of all randomized subjects who consumed at least one dose of the nutritional intervention. At visits V_1_–V_3_, subject compliance was evaluated by the site staff through the parental report in the diary and by the evaluation of the product left. Compliance was assessed in percent of actual duration compared to the planned duration. Values above 80% for iPad-based training on an expected total of 78 days of such training and above 90% for product intake relative to a daily intake were considered as compliant. Based on the compliance data, a separate per-protocol (PP) dataset was defined and used to assess the sensitivity of the results observed in the primary analysis set. Statistical analyses were performed on differences between outcome measures at V_3_ and baseline (V_3_–V_0_). A one-way ANCOVA was conducted to assess the difference between the experimental and the control group for WPPSI-IV, CBCL 1.5–5, and PICCOLO scores. Normality assumptions for V_3_–V_0_ were confirmed by using QQ plots and Shapiro–Wilk test for normality. The ASQ-3 scales were analyzed using the generalized estimating equation (GEE) model, a semi-parametric approach to longitudinal analysis on non-normally distributed data. The 12 parent diary questions we compared between the two groups at each visit using either a chi-squared test for categorical responses or a *t*-test for continuous responses. Model-based estimates were controlled for gender and baseline scores. Comparison between groups of the incidence of adverse events was done by Fisher’s exact test. Descriptive statistics in the figures report mean outcome measures and standard error per group and time point. Statistical analysis was performed using SAS 9.3.1 (www.sas.com) on a 5% significance level.

## 3. Results

The experimental and control groups were well matched with respect to the screening measure of the home stimulation, cognitive functioning and development, as well as baseline nutritional status (Table 2, Table 3 and Table 4). The majority (96.4%) of children involved in the study was categorized as being above the normal range of cognitive development as indicated by the Ages and Stages Questionnaire. On average, children’s dietary intake based on a 24-h recall at baseline fell below 77% of the Recommended Dietary Allowance (RDA) for children aged 3–5 years for calcium, carbohydrate, energy, fat, and Vitamin A (https://ods.od.nih.gov/). However, children’s growth measures and their nutritional status assessed by means of the Hydroxyproline Index were in the healthy range. The Hydroxyproline Index was well below the reference value of 46 nmol/mg (https://www.mayomedicallaboratories.com/test-catalog/Clinical+and+Interpretive/60475). Per inclusion criteria, children’s weight relative to their height was within the normal range according to the WHO. Weight, height, nutritional status, and outcome measures were compared between the two intervention arms at V_0_. For weight and height, this was done separately for both males and females. No statistically significant differences were observed at V_0_ (all *p*-values > 0.05, Table 2, Table 3 and Table 4).

Compliance with the study protocol based on parental reports was high, with 91.7% of the participants being compliant regarding product intake and 93.7% of the participants being compliant regarding iPad use. The compliance measures were consistent with the median amount of iPad playing time recorded per week (mean = 32 min, Table 5). The reported use of paper-and-pencil learning material was 1.26 h per day (Table 5).

**Figure 2:**
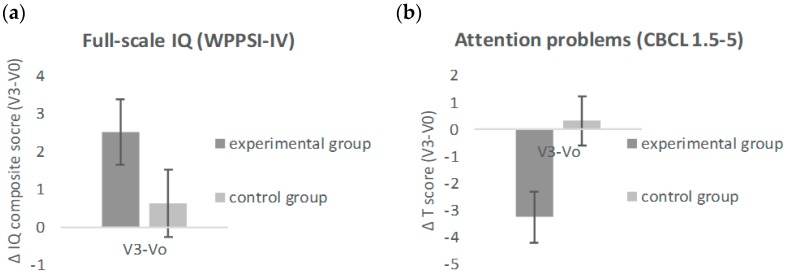
Cognitive and behavioral differences between experimental and control groups expressed as change from baseline after the 6-month intervention. (**a**) Full-scale IQ composite score and (**b**) attentional problems are plotted separately for the experimental and the control groups. Error bars reflect standard errors.

After the intervention, children in the experimental group showed a larger increase in cognitive performance and a larger reduction in attentional problems compared to the control group (Figure 2). That is, the increase from baseline in full-scale IQ composite score derived from the WPPSI-IV was larger for children in the experimental group (LS Mean = 2.82, SE = 0.80) compared to children in the control group (LS Mean = 0.57, SE = 0.79, *p* < 0.05, 95% CI (0.02, 4.47)). This significant result obtained in the primary analysis set was confirmed in the per-protocol dataset as a trend (*p* < 0.10). None of the IQ subscores showed significant differences between the two groups. Parents reported a significantly larger reduction in attention problems as measured by the CBCL 1.5–5 for children in the experimental group (LS M = −2.97, SE = 0.77) compared to children in the control group (LS M = −0.13, SE = 0.76, *p* < 0.05, 95% CI (−4.98, −0.70)). This effect obtained in the primary analysis set was confirmed in the per-protocol dataset.

Relative to the baseline measure, a larger increase in time spent playing with the child at home per day was reported by the parents of control group compared to the parents of the experimental group both for V_1_ and V_3_ (*p* < 0.05). Across all time-points, parents in the control group spent 2.5 h and the parents of the experimental group spent 2.0 h per day playing with their child (Table 5).

No other significant group differences were observed in the full analyses set on nutritional and anthropomorphic measures or other behavioral and cognitive measures. Namely, the nutritional status of the participants was evaluated at baseline and at the end of the intervention and the results show no statistically significant difference between the two intervention arms on change from baseline for creatinine (*p*-value = 0.89) and hydroxyproline (*p*-value = 0.53) (Table 4). Weight and height of the study participants were compared between the two intervention arms at V_3_ for both males and females and no statistically significant difference was observed for any of them (all *p*-values > 0.05, Table 3).

There was a significantly higher rate of infectious-related symptoms reported in the experimental compared to the control group during the time-course of the intervention, particularly fever (53.7% of subjects in the experimental group versus 38.1% in the control group *p* < 0.04), cough (38.9% of subjects in the experimental group versus 22.7% in the control group, *p* < 0.05), and infections (66.3% of subjects in the experimental group versus 47.4% in the control group, *p* = 0.01). Upper respiratory infectious conditions were the predominant contributor to these differences.

## 4. Discussion

This study investigated cognitive and behavioral benefits of a combined nutritional and cognitive stimulation program on children with below-average psychosocial stimulation in their home environment. All children were at preschool age and with cognitively normal development. The children in the experimental group compared to the control group increased their full-scale IQ, compared to the baseline measure, as a result of the intervention. Moreover, children of the experimental group compared to the children in the control group showed a more pronounced decrease in parent-reported attentional problems compared to the baseline measure. In both groups, the intervention substantially fostered parent–child interaction.

Cognition and attention-related behavior in preschool aged children in Indonesia significantly improved after the children received a combination of nutrient supplementation and cognitive stimulation. Such combined interventions are widely considered the gold standard for interventions in developing countries [1]. Due to the design of the study, the observed beneficial effect of the intervention can only be interpreted as a consequence of both the nutritional and cognitive intervention and their relative contributions remain unknown. Here, we evaluate the effects of intervention program on cognition and behavior and discuss the factors that may have contributed to these benefits.

Nutritional interventions in previous studies in early childhood included either nutritional advice to caregivers [11] or supplementation [11,12,16,17]. In our study, nutritional supplementation comprised nutrients that have specifically been associated with cognitive functioning in other dietary supplementation studies. These include alpha-linolenic acid (ALA), the metabolite of which, docosahexaenoic acid (DHA), is associated with faster response times on attention tasks and vocabulary [28,29] and several of the B vitamins, of which namely B6 was associated with scholastic achievement [30]. Moreover, the supplementation comprised the micronutrients zinc, magnesium, and iron. The nutrient intake of both zinc and magnesium was previously found to correlate positively with scholastic achievement [30] and blood levels of zinc and iron were associated with verbal abilities [31]. Compliance with the product intake was relatively high, with 176 children out of 192 taking the product as indicated. The nutritional component of our combined intervention likely provided the children with the nutrients they needed to profit optimally from the psychosocial stimulation aspect of the intervention.

We did not observe any influence of the intervention on the growth of the children during the 6 months of the intervention. Across both groups, children grew on average 3.4 cm and their weight increased by 1.2 kg. While some earlier nutritional intervention studies failed to find growth differences [4,15], others reported increased growth after nutritional supplementation [10,14]. The latter studies tested a similar number of participants as our study. However, these studies included children that were undernourished. The lack of growth differences between the two groups in our study could also be explained by the matched amount of energy intake in the experimental and the control group, as both the fortified and unfortified milk powder provided a similar amount of kcal. Importantly, whether or not nutritional supplementation provides a benefit in growth also depends on the growth status of the children at baseline. In our study, there was no indicator for malnutrition. Thus, it comes as no surprise that fortification did not influence the growth of the children.

Global cognitive functioning, as measured by WPPSI, increased during the intervention to a greater extent in the children of the experimental group compared with the control group. While this result is in line with previous studies reporting effects on cognition in young children in developing countries after a combined intervention of nutrition and cognitive stimulation [10,11,12,15], our study adds to the previous body of evidence. The above mentioned studies included children that were malnourished, while the children participating in our study where normally developing. Children’s weight per age and their Hydroxyproline Index indicate an absence of malnutrition. Moreover, both a parental assessment and the DIKNAS test categorized all children as normal or above normal in their cognitive development. The observed effects in our study, although robust, were relatively small regarding both the cognitive (standardized effect size = 0.15) and attention (standardized effect size = −0.2) measures. This could in part be explained by the fact that the children were normally developing. Thus, our study indicates that cognitive stimulation in combination with nutritional supplementation could positively influence global cognitive functioning even in healthy and normally developed children.

The cognitive benefits of the intervention were not specific to one cognitive domain but reflected an increase of full-scale IQ. Although the highest effect sizes were observed in the verbal comprehensions scores, there was not one submeasure of IQ that drove the improvement in IQ in the experimental group. The cognitive intervention comprised two components: the iPad-based tasks and the learning activities performed at home. Children were highly compliant with both aspects of the cognitive intervention. They spent, on average, more than an hour per day on the learning activities and on average 32 min per week on the iPad-based tasks. The ASQ Learning Activities focused on communication, gross and fine motor skills, problem solving, social interaction, and early language skills. In contrast, the iPad tasks were designed to stimulate memory, language, psychomotor skills, problem solving, and attention. Of these cognitive abilities, namely attentional control, working memory, and problem solving have previously been associated with general intelligence [38]. Thus, the cognitive intervention used in this study targeted a broad range of cognitive abilities. As there was no disproportional increase in ability with regard to one specific sub-scale of IQ, we assume that the intervention program provided the intended broad and comprehensive stimulation. The practical implication of our result is that a holistic psychosocial stimulation in early childhood intervention programs is beneficial for a child’s cognitive development.

Next to the abovementioned aspects of the cognitive stimulation, the observed beneficial effects could have been influenced by the interaction between parents and children. Although parents of the control group spent more time playing with their children, when considering the playing time and the time spent on the psychosocial stimulation part of the intervention, the parents of the experimental group compared to the control group spent more total time interacting with their children. Both the learning materials and the iPad-based tasks fostered communication between the caregiver and the child. While there is no data on the quality of the parent–child interaction and no qualitative differences were observed in this interaction based on the PICCOLO checklist, previous studies suggest that the mere time parents spent playing with the child likely had a beneficial influence by fostering attachment security (for a review, see [39]). Not only does time spent in joint activities with a caregiver correlate with more happiness in children [40,41], joint activities help caregivers to better understand the child, which, in turn, facilitates enduring relationships [42,43]. Resulting increases in attachment security are linked to improved cognitive functioning, including working memory and cognitive flexibility [44]. Thus, the parent–child interaction induced by the cognitive stimulation in our study might have played a role in driving the positive effects of the intervention.

Compared to the time before the intervention, parents of the control group reported a larger increase in time spent playing with the child at home compared to the parents of children in the experimental group. As the caregivers in the experimental group also engaged in the iPad-based tasks, the total time spent interacting with the child was still higher in the experimental group compared to the control group. It is, however, remarkable that the caregivers in the control group increased the time they spent interacting with the child. It is possible that this is due to the Hawthorne effect [45,46,47,48]. Parents of the control group filled in a questionnaire regarding how much time they spent playing with the children. While parents might have already been well aware that time spent playing with children fosters the wellbeing of the children, being required to fill in a related questionnaire might have reactively increased the time they spent playing with the child. For parents of the experimental group who already spent time coming to the training intervention and spent a higher total time with the child than the parents of the control group, we might observe a ceiling effect in that the parent did not have more time available to spend with the child. Thus, the Hawthorne effect potentially modified the behavior of the caregiver and increased the interaction between caregiver and child.

This is the first study that combines a nutritional intervention with iPad-based cognitive stimulation and offers valuable information on the potential use of digital stimulation programs in young children. In order to avoid potential negative effects of screen-use in young children, such as decreased short-term memory skills and social skills [49,50,51], screen time was limited to a maximum of 15 min per day. Moreover, the focus of the tasks, namely in the reading and problem-solving tasks, was on parent–child interaction. Other researchers have suggested similar approaches of combining face-to-face interaction with screen-based stimulation [52]. Data on compliance with the program as well as on the use of the iPad-based activities indicate the feasibility of such an intervention. Interestingly, parental involvement required by the iPad-based tasks did not seem to replace other playing activities between the parent and the child. Parents of the children in the experimental group still spent a significant amount of time playing with their children. This evidence suggests that sensibly designed iPad-based interventions that are put into a social context could be an effective way of providing cognitive stimulation even to young children.

One of the limitations of this study is the lack of an experimental condition comprising the nutritional and the psychosocial stimulation alone. Consequently, when interpreting the cognitive and behavioral benefits observed in the experimental group, we cannot differentiate between the contribution of either the nutritional or the behavioral intervention alone nor can we know whether an interaction of the two interventions was driving the effect. The rationale for a combined intervention was that it is the state of the art in early childhood interventions. Any potential nutritional deficits in children are reduced and potentially compensated by the nutritional intervention, thus providing the children with the nutrients they need to profit optimally from the psychosocial stimulation aspect of the intervention. Further studies will need to address the differential benefits that can be derived from nutritional and behavioral interventions, respectively. Moreover, the generalization of the study is limited to the geographic and age distribution of the children tested in the study.

We did observe a higher rate of infectious-related symptoms in the experimental group compared to the control group mainly due to respiratory infectious conditions. Based on the nature of the infection and similar observations reported in the literature [53,54,55], our clinical assessment is that the increased infection rate is likely not linked to the nutritional intervention but rather the result of a potentially increased exposure to pathogens because of the attendance to the community centers. In our study, only children assigned to the experimental group visited community centers 3–5 days per week, while children of the control group only visited the centers for the assessments at V_0_–V_3_. While we cannot discard the possibility that the increased rate of infectious-related symptoms is the result of sharing the electronic devices amongst children, a number of studies demonstrated that daycare attendance significantly increases the risk of infections in preschool children [56]. Future studies should pay special attention to implement hygiene protocol when devices are shared amongst children.

We assessed potential biases in our studies by means of the Cochrane risk of bias assessment tool [57]. We have excluded a potential selection bias in that assignment was controlled for age and gender using a standard minimization method. Although not controlled by minimization, this randomized assignment of the children ensures minimal difference in other participant characteristics between the two groups. This is readily visible from the mean and standard deviation measures reported in Table 2, Table 3 and Table 4. Moreover, the allocation sequence was concealed to the experimenter in that the assignment was nonobvious and the study product code concealed the nature of the intervention. The following potential biases were evaluated with regard to the critical study outcome, the WPPSI. Performance bias was addressed by appropriate blinding of participating children, parents, and study personnel to the nature of the nutritional intervention. While the psychologist assessing the outcome was blind to the training intervention, we cannot guarantee the blinding of the participants with regard to the other training condition. However, parents and children were not intentionally informed about the two training conditions. To control for detection bias, the WPPSI was assessed by a tester who was blind to the experimental condition to which the child had been allocated. The statistical analysis plan was defined in a blinded manner before breaking the code. Eight participants (three and five per group, respectively) dropped out of the study because they either did not consume at least one dose of milk powder or dropped out before the allocation to an experimental condition. Thus, there was no systematic error caused by an unbalanced loss of patients across the two intervention groups, which rules out an attrition bias. We have reported all the assessed measures and can, thus, exclude a reporting bias.

## 5. Conclusions

Early childhood intervention combining nutritional supplementation with cognitive training that focuses on the interaction between caregiver and child could have a significant positive effect on general cognitive abilities and parental ratings of attention problems in preschool-aged children.

## Figures and Tables

**Table 1:** Composition of fortified milk powder and skimmed milk (control).

Macronutrients	Unit	Control Product (Skimmed Milk Powder)	Fortified Milk Powder
Energy	kcal/100 g	467.8	477.7
	kcal/serving	168.4	172
Fat	g/100 g	17.8	21.4
	g/serving	6.4	7.7
Alpha-linolenic acid	mg/100 g	60.9	556.6
	mg/serving	28.1	200.4
Protein	g/100 g	14.8	16.3
	g/serving	5.3	5.9
Fortification	mg/100 g	none	Zinc (8), Iron (11.4), Magnesium (141.7), Thiamin (1.05), Niacin (11), Pyridoxine (1.77), Biotin (0.0177), Vitamin C (97.3), and Alpha-linolenic acid (ALA, 556.6)

**Table 2:** Participant demographics, environmental characteristics, cognitive functioning, and development. Mean and standard deviations are indicated. No significant differences were observed between the two groups (*p* > 0.05).

Baseline Measure	Experimental Group (*n* = 95)	Control Group (*n* = 97)
Age (years)	3.90 ± 0.57	3.93 ± 0.57
Sex (f/m)	45/60	45/52
HOME Inventory (score)	21.14 ± 3.34	21.15 ± 3.25
WPPSI (full-scale IQ score)	81.64 ± 9.937	80.51 ± 10.294
Ages and Stages (above/within normal)	93/2	92/5

**Table 3:** Participant nutritional status as measured by anthropometric measures (height and weight) at baseline (V_0_) and V_3_ indicated separately for gender and experimental group by mean and standard deviation. No significant differences were observed between the two groups at all time-points (*p* > 0.05).

	Treatment Group				Control Group			
	Male		Female		Male		Female	
	V_0_	V_3_	V_0_	V_3_	V_0_	V_3_	V_o_	V_3_
Height (cm)	99.05 ± 6.332	102.11 ± 6.429	99.54 ± 5.733	103.32 ± 5.593	100.05 ± 5.151	103.67 ± 5.203	98.30 ± 5.572	101.56 ± 5.400
Weight (kg)	14.84 ± 2.212	15.83 ± 2.797	14.92 ± 2.664	16.48 ± 2.969	15.35 ± 2.047	16.39 ± 2.406	14.21 ± 2.034	15.49 ± 2.231

**Table 4:** Participant nutritional status at baseline (V_0_) and V_3_ as measured by the Hydroxyproline Index in urine samples. Mean and standard deviations are indicated. No significant difference were observed between the two groups at all time-points (*p* > 0.05).

	Experimental Group		Control Group	
	V_0_	V_3_	V_0_	V_3_
Hydroxyproline (ug/uL)	0.1629 ± 0.09821	0.6146 ± 0.29840	0.1572 ± 0.07314	0.6446 ± 0.31704
Creatinine (mg/dL)	87.49 ± 53.575	98.29 ± 46.407	86.34 ± 46.975	99.30 ± 50.867
Hydroxyproline Index: Hydroxyproline/creatinine (nmol/mg)	1.48	5.0	1.51	5.2

**Table 5:** Cognitive stimulation including (a) the use of iPad-based tasks by children of the experimental group as recorded on iPads, (b) the use of paper–pencil learning material (ASQ learning activity) based on parental report, and (c) additional time spent by parents playing with the child outside of experiment-related playing activities based on parental report. Time is indicated for each group of participants between V_0_ and V_3_ per day. Mean and standard error are reported. * indicates significance.

		Experimental Group	Control Group	*p*-Value (Diff. between Groups)
(a) Use of iPad-based tasks (minutes/week)	V_0_ to V_1_	32.9	-	-
	V_1_ to V_2_	34.8	-	-
	V_2_ to V_3_	29.9	-	-
(b) How long the child played with the ASQ learning (h/day)	V_0_ to V_1_	1.26 (0.09)	-	-
	V_1_ to V_2_	1.08 (0.07)	-	-
	V_2_ to V_3_	1.43 (1.11)	-	-
(c) How much longer the parents played with the child compared to before the study (h/day)	V_0_ to V_1_	2.26 (0.16)	2.89 (0.21)	*p* < 0.05 *
	V_1_ to V_2_	1.95 (0.28)	2.56 (0.21)	*p* = 0.08
	V_2_ to V_3_	1.54 (0.13)	2.19 (0.21)	*p* < 0.05 *
